# Notes from “The Mayo Clinic”

**Published:** 1905-08

**Authors:** C. M. Rosser

**Affiliations:** Dallas; Rochester, Minn.


### Clinical Notes.

### Notes From "The Mayo Clinic."

BY C. M. ROSSER, M. D., DALLAS.

*Editor Texas Medical Journal.*

DEAR DR. DANIEL: As promised, I send you a few notes from observation which may interest your readers. "The Mayos" are three: Dr. W. W. Mayo, the eldest, a pioneer practitioner in Minnesota; Dr. W. J. Mayo, President-elect of American Medical Association, and Dr. C. H. Mayo, two years his junior, now 40. Rochester, the home of all, and the birth place of the latter, claims only 8000 inhabitants. Besides, with the disproportion of clinic and city, visitors are impressed by the courtesy of surgeons, associates and assistants, the clinical care in diagnosis and in pathological confirmation, the width of surgical conditions covered, the simplicity of detail methods and uniformity of results. St. Mary's Hospital of 180 beds is for surgical cases only. Report for the year 1904 shows: Total number of operations 3131, total number of patients 2501, number of in patients 2093, number of out patients 408. Total death rate, intra-peritoneal, non-malignant, two and seven-tenths per cent. This average of ten operations daily for the year 1904 was exceeded by a rate of from twelve to fifteen daily during the past thirty days.

*Chronic and Intolerable Neuritis of Fifth Nerve.—Types:* Confined to one branch and distributed. Infra-orbital most common. Hesitates to remove Glasserian ganglion except as *dernier ressort.* Mortality one in twelve. Less radical operation preferable for partial or temporary relief, which is some times total and permanent. Injection method (osmic acid) discussed by a London writer, Murphy of Chicago and others, has been successful in some instances, but is yet under trial.

*Case:* (Operations by Thiersche's Method of Torsion.) Through small incision nerve is grasped by slender forceps, twisted moderately tight by rotating in each direction, then by rolling on forceps as by windlass, proximal end makes traction against peripheral. Tendency to return by regeneration (one year or less) is overcome by placing silver screw in the infra orbital foramen which permanently blocks and prevents regeneration of nerve. (Stress is laid upon the necessity of removing gustatory nerve also as frequently it is a point of excitation.) Gustatory is easily found by traction on the tongue as a cord-like feeling on the finger from jaw. Mucous membrane is pierced and nerve hooked up and held. Inferior maxiliary is now reached by incision within mouth by cutting mucous membrane over ramus in line with last molar tooth, and the spine which marks entrance of inferior dental canal. Pass strabismus hook beneath and lift inferior maxilary nerve. Gustatory nerve already demonstrated is now retracted and both caught by forceps then removed by torsion. The nerve comes out as a brush-like tail, which floats in water as fine filaments. Patients are kept in bed and are detained only a few days.

*Dissection of Neck Glands Important Point.*—Unnecessary to sever sterno-cleido mastoid muscle. Incision posterior to it. Gause packed under omo-hyoid, to distend jugular vein, marking it for sight, and if wounded, to prevent from the dangerous accident of sucking air. Sensory branches of cervical plexus may be sacrificed, but care must be exercised not to wound spinal accessory nerve, the loss of which induces atrophy of shoulder, and in young adults may cause lateral curvature. Make thorough dissection, removing all lymph nodes.

*Empyema.*—Usual resection. No irrigation. Drainage by spiral split rubber tube, each end out, avoiding irritation to lung, as occurs when tube ends are left inside cavity. Unfavorable cases, after some months, require removal of all ribs covering pathological cavity, and incision of the pleura which binds the lungs. Then leave skin covering only.

*Thyroidectomy for Tumor of Trachea.—Case:* Boy; 6 years; loss of voice several months and total; later interference with respiration.

*Operation:* "T." incision for low tracheotomy made. Cocaine solution to control spasmodic reflexes interfering with respiration. Upper portion packed with gauze to prevent hemorrhage, tube inserted as usual and held by tape. Thyroid cartilage opened by transverse incision near upper border, exposing tumor mass, vertical incision between vocal cords allowing removal of tumor with knife and curette. Carbolic acid applied to raw surface, followed by alcohol; wound closed by cat gut sutures, tube removed on second day; pathological report—Papilloma.

*Refilling Bone Cavities.*—Case*:* Old osteo-myelitis of femur. Usual removal of necrotic tissue; cleanse and dry. Use bone plug common to Morehoff's Clinic, consisting of iodiform 60 parts, spermaceti 40 parts, oil of sesame 4 parts. When poured into cavity hot, forms solid plug to be replaced gradually by granulation tissue.

*Hydrocele and Varicocele.*—Incise tunica vaginalis and invert *sac permanently.* If veins are excessive, resect in the usual way and bring together. In hydrocele nothing is needed except that testacle and other scrotal contents are left covered only by skin.

*Internal Shortening Round Ligaments.*—(C. H. Mayo method.) Formerly used operations described by Ferguson, Simpson, Noble and others, but now applies the principles of these as follows: Median incision, usual. Dissect skin laterally, one and one-half inches. Incise fascia. Pass Cleveland ligature carrier above and around rectus muscle to about internal ring; enter abdominal cavity and pass along around ligament, extra peritoneal, to within one and one-half to two inches of uterus; grasp round ligament with carrier point, and retract to point of incision in fascia, where ligament is stitched with linen or cat gut. Other side the same.

*Gall Bladder Disease. Gall Stones.*—Many people have gall stones without classical symptoms as pain in bladder region, jaundice and passage of stones. Symptoms are frequently referred to the stomach as in chronic dyspepsias, gastric neuralgias, etc. Important to make early diagnosis. Mortality where infection is confined to gall bladder one-half of one per cent, as in internal appendectomies. Common duct cases ten per cent and with pancreatic and liver involvements as high as thirty per cent. Complete removal of gall bladder is practiced in from two to five cases on an average as they come. When duct drainage is necessary, as a rule, prefer the common duct rather than the hepatic. Rubber tube wrapped with gauze, around which is placed rubber tissue, is inserted in bladder or duct and held by purse-string suture, to be removed in from four to eight days.

*Gastro Enterostomy.*—Recommended and practiced as a relief measure and sometimes cure of conditions obstructing pylorus and causing some stomach dilation. Results now justify it as an operation of selection in intractable ulcer of the stomach or duodenum, and for carcinomata of this region. Magraw ligature is not used and the Murphy button but seldom. An anastomosis between posterior stomach and illium is made by delicate suturing so well described by W. J. Mayo and others in recent literature. (*Annals of Surgery,* December, 1904.) This method secures drainage of the obstructed or otherwise dilated stomach, relieves ulcerated surfaces and permits overstretched stomach structures to regain their tonicity. It is in this field, and indeed the entire upper abdomen, that the most brilliant results are now being obtained, but I fear I have already taxed the proper limit of space for this letter, and must omit notes upon many important matters.

Rochester, Minn., July 27, 1905.

